# Evolutionary Dynamics of the *Pgk1* Gene in the Polyploid Genus *Kengyilia* (Triticeae: Poaceae) and Its Diploid Relatives

**DOI:** 10.1371/journal.pone.0031122

**Published:** 2012-02-20

**Authors:** Xing Fan, Li-Na Sha, Jian Zeng, Hou-Yang Kang, Hai-Qin Zhang, Xiao-Li Wang, Li Zhang, Rui-Wu Yang, Chun-Bang Ding, You-Liang Zheng, Yong-Hong Zhou

**Affiliations:** 1 Triticeae Research Institute, Sichuan Agricultural University, Sichuan, People's Republic of China; 2 Key Laboratory of Crop Genetic Resources and Improvement, Ministry of Education, Sichuan Agricultural University, Sichuan, People's Republic of China; 3 College of Resources and Environment, Sichuan Agricultural University, Sichuan, People's Republic of China; 4 Department of Biology and Science, Sichuan Agricultural University, Sichuan, People's Republic of China; American Museum of Natural History, United States of America

## Abstract

The level and pattern of nucleotide variation in duplicate gene provide important information on the evolutionary history of polyploids and divergent process between homoeologous loci within lineages. *Kengyilia* is a group of allohexaploid species with the StYP genomic constitutions in the wheat tribe. To investigate the evolutionary dynamics of the *Pgk1* gene in *Kengyilia* and its diploid relatives, three copies of *Pgk1* homoeologues were isolated from all sampled hexaploid *Kengyilia* species and analyzed with the *Pgk1* sequences from 47 diploid taxa representing 18 basic genomes in Triticeae. Sequence diversity patterns and genealogical analysis suggested that (1) *Kengyilia* species from the Central Asia and the Qinghai-Tibetan plateau have independent origins with geographically differentiated P genome donors and diverged levels of nucleotide diversity at *Pgk1* locus; (2) a relatively long-time sweep event has allowed the *Pgk1* gene within *Agropyron* to adapt to cold climate triggered by the recent uplifts of the Qinghai-Tibetan Plateau; (3) sweep event and population expansion might result in the difference in the d_N_/d_S_ value of the *Pgk1* gene in allopatric *Agropyron* populations, and this difference may be genetically transmitted to *Kengyilia* lineages via independent polyploidization events; (4) an 83 bp *MITE* element insertion has shaped the *Pgk1* loci in the P genome lineage with different geographical regions; (5) the St and P genomes in *Kengyilia* were donated by *Pseudoroegneria* and *Agropyron*, respectively, and the Y genome is closely related to the Xp genome of *Peridictyon sanctum*. The interplay of evolutionary forces involving diverged natural selection, population expansion, and transposable events in geographically differentiated P genome donors could attribute to geographical differentiation of *Kengyilia* species via independent origins.

## Introduction

Duplication is a prominent feature of plant genomic architecture. Genome duplication or polyploidy provides a reservoir of duplicate genes as substrates for potential evolutionary innovation [1]. Analysis of the levels of diversity and the patterns of substitution in duplicate gene not only traces evolutionary history of polyploids [2], but also provides insight into how the evolutionary process differs between lineages and between homoeologous loci within lineages [3], [4]. Theoretical and empirical investigation suggested that the diversity of duplicate gene is unlikely equivalent, and may arise from various forms of natural selection [3], [5]–[7], population size and history [8], introgression [9], mating system [10], recombination [11], mutation rate [6], and gene conversion [12]. It has been reported that transposable element indels shaped the homoeologous loci, which was responsible for the patterns of diversity of duplicate gene [13]. In addition, forces acting on the levels and patterns of diversity also arise from the domestication bottlenecks [14]. Therefore, differences in the levels and patterns of nucleotide diversity of duplicate gene may reflect numerous forcing factors. To segregate the effects of various forcing factors, it is necessary to obtain evolutionary dynamic data from additional homoeologous loci within a given phylogenetic framework [3].

*Kengyilia* Yen et J. L. Yang, a polyploid perennial genus in the wheat tribe (Poaceae: Triticeae), includes about 22 perennial species distributed in a different range of natural habitats over the upper and middle mountain ranges of Central Asia and the Qinghai-Tibetan Plateau [15]. Cytogenetic evidence suggested that *Kengyilia* species arose from two hybridization events followed by genome doubling of three ancestral diploid species with different genomes St, Y and P [15]–[19]. The St and P genomes are derived from *Pseudoroegneria* (Nevski) Á Löve and *Agropyron* Gaertn., respectively [20]. It is unknown where the Y genome originates, although it is a fundamental *Kengyilia* genome [19]. Dewey [21] considered that the Y genome has its origin in Central Asia or the Himalaya region, and may be extinct. Analysis of some StY genome species using β-amylase gene sequences yielded distinct presumed Y-genome starch synthase sequences [22]. Based on ITS sequence analysis, Liu et al. [23] suggested that the Y genome might originate from the St genome. However, data presented by Sun et al. [24] suggested that the Y genome is sister to the W and P genomes. Therefore, the origin of Y genome is open for further study.

Previous studies based on RAPD (Random amplified polymorphic DNA polymorphism) [25], RAMP (Random Amplified Microsatellite Polymorphism) [26], C-banded karyotypes [27], and ITS sequence [28] suggested that the pattern of evolutionary differentiation of *Kengyilia* species associated with geographical origin from Central Asia and the Qinghai-Tibetan plateau. Zhou et al. [25] speculated that the pattern of evolutionary differentiation of *Kengyilia* species might genetically arise from its parental lineages with two different geographical origins (Central Asia and The Qinghai-Tibetan plateau). Based on the cytogenetic and geographic data, Yen et al. [19] hypothesized that the biological factors from diploid *Agropyron* (P genome) species might play an important role in influencing the genetic differentiation of *Kengyilia* species. While these studies add to our understanding of phylogeny and genetic differentiation of *Kengyilia*, little is known about the evolutionary forces acting on the geographical differentiation of *Kengyilia*, and further information on whether the biological factors from the P genome influences the patterns of genetic diversity of *Kengyilia* species is still outstanding.

Phosphoglycerate kinase (Pgk1), a key ATP-generating enzyme in the glycolytic pathway, catalyzes the conversion of 1, 3-diphosphoglycerate to 3-phosphoglycerate. Analysis of the *Pgk1* gene showed that it is present as a single copy per diploid chromosome in grass [29]. The *Pgk1* gene has been successfully used to study the phylogeny and evolutionary history of *Triticum/Aegilops* complex [30], [31]. In this study, three homoeologous copies the *Pgk1* gene were isolated from each the fifteen sampled *Kengyilia* species and analyzed with those from 47 diploid taxa representing 18 basic genomes in Triticeae. The objectives were to: (1) document the patterns of molecular evolutionary divergence among homoeologues of the *Pgk1* gene in hexaploid StYP *Kengyilia* and between polyploidy and its diploid genome donor; (2) determine whether the patterns of *Pgk1* sequence variation within the P genome lineages reflects the geographical differentiation of *Kengyilia* species; (3) explore evolutionary forces acting on the *Kengyilia* species with different geographical region; (4) identify the possible origin of the Y genome.

## Materials and Methods

### Taxon sampling

Fifteen *Kengyilia* species were included in this study and were analyzed together with 47 diploid taxa representing 18 basic genomes in the tribe Triticeae (Table S1). *Pgk1* sequences for 9 accessions representing the S, D, I, R and A genomes were obtained from published data [30]. The remaining *Pgk1* sequences are new data and have been deposited in GenBank. *Bromus inermis* L. was used as outgroup. The seed materials with PI and W6 numbers were kindly provided by American National Plant Germplasm System (Pullman, Washington, USA), while the seed materials with ZY and Y numbers were collected by ourselves, which no specific permit is required. The plants and voucher specimens are deposited at Herbarium of Triticeae Research Institute, Sichuan Agricultural University, China (SAUTI).

### DNA amplification, homoeologous sequence isolate, and sequencing

DNA extraction followed a standard CTAB protocol [32]. The *Pgk1* gene was amplified with the Pgk1-specific primers PgkF1 (5′-TCGTCCTAAGGGTG TTACTCCTAA-3′) and PgkF2 (5′-AAGCTCGCGCCACCACCAGTTGAG-3′). PCR was conducted under cycling conditions reported previously [29]. PCR products were cloned into the pMD18-T vector (TaKaRa) following the manufacture's instruction.

PCR amplicons of single-copy nuclear genes from allopolyploid species will produce a heterogeneous mix of homoeologues. To separate the homoeologues of the *Pgk1* gene from each *Kengyilia* accession, we performed the following process. Firstly, approximately 30 positive clones from each accession were screened by direct PCR using primer PgkF1 and M13R (on the side of the cloning site in the plasmid). Secondly, St-type (5′-GGTATTCTTGTGTTCCACACCA-3′) and P-type (5′-ATCZAGACYTCTAATCAAGCA-3′) Pgk1-specific primers were designed and used each together with the reverse primer PgkF2 to screen the St- and P-type *Pgk1* sequences from above 30 positive clones with *Pgk1* inserts, respectively. The positive clones containing the Y-type *Pgk1* sequences were also obtained. The cloned PCR products were commercially sequenced in both directions by TaKaRa Biotechnology Co. Ltd. (Dalian, China), and an additional internal primer (5′-GATGGAGCTGTTTCAAACC-3′) was used to sequence the internal portion of the cloned PCR products. All the sequences from *Kengyilia* species were determined based on at least five independent St-, Y- and P-type clones, respectively.

### Data analysis

Multiple sequences were aligned using ClustalX [33] followed by manual adjustment. To reduce the size of the matrixes and the possible impact of PCR artifacts, unique substitutions in single clones were ignored and several identical sequences were represented by a single sequence in alignments. Following an initial phylogenetic analysis, the number of sequences used for alignment was reduced by keeping only one sequence if more sequences of the same accession formed a monophyletic group.

To assess the divergence and genetic relationships between allopolyploids and its diploid progenitors, nucleotide diversity was estimated by Tajima's π [34], Watterson's θ [35], the number of fixed differences (S_F_) and the numbers of shared polymorphisms (S_S_) [36]. Tests of neutrality including Tajima's, and Fu and Li's D statistic were performed as described by Tajima [34], and Fu and Li [37]. Significance of D-values was estimated with the simulated distribution of random samples (1000 steps) using a coalescence algorithm assuming neutrality and population equilibrium [38].

To detect selective constraints on the coding portions (the introns were excluded) of the homoeologous *Pgk1* gene, the ratio of nonsynonymous to synonymous substitution (d_N_/d_S_) were computed using the modified Nei-Gojobori method in MEGA 4.0 [39] and the single likelihood ancestor counting (SLAC) approach implemented by the Datamonkey analysis [40]. In the modified Nei-Gojobori analysis, the significance of difference between *d*_N_ and *d*_S_ was estimated using the Z statistics, with standard errors based on 1000 bootstrap replicates using MEGA 4.0 [39]. In SLAC analysis, a 95% confidence interval (95% C.I.) for the ratio of *d*_N_ to *d*_S_ was estimated using profile likelihood [40]. We also performed the McDonald–Kreitman (1991) test on the coding portions of the homoeologous *Pgk1* gene using DnaSP 4.10.9 [41]. Significance of the test was determined by a Fisher exact test [42].

Phylogenetic analyses were conducted using maximum likelihood (ML) and Bayesian inference (BI). The evolutionary model used for the phylogenetic analysis was determined using ModelTest v3.0 with Akaike information criterion (AIC) [43]. The optimal model identified was GTR+G+I. ML analysis was performed using PAUP*4.0b10 (Swofford D L, Sinauer Associates, http://www.sinauer.com). ML heuristic searches were performed with 100 random addition sequence replications and TBR branch swapping algorithm. The robustness of the trees was estimated by bootstrap support (BS) [44]. BI analysis was performed using MrBayes v3.0 [45]. Four MCMC (Markov Chain Monte Carlo) chains (one cold and three heated) were run for 1,000,000 generations. The first 2500 trees were stationary discarded as “burn-in”. The remaining trees were used to construct the 50%-majority rule consensus trees. The statistical confidence in nodes was evaluated by posterior probabilities (PP).

Clock-like evolution of *Pgk1* sequences within *Kengyilia* and its putative diploid species was evaluated with a likelihood ratio test comparing the likelihood scores from the unconstrained and clock-constrained analyses, implemented in PAUP*4.0b10. Substitution rates were significantly heterogeneous (χ^2^ = 173.44, df = 67, P<0.0001), implying a very poor fit to the molecular clock. Therefore, divergence times with 95% confidence intervals were estimated using Bayesian relaxed molecular clock method, implemented in BEAST v1.4.6 [46]. The lack of fossils for Triticeae precluded a direct calibration of tree topologies. Instead, molecular dating was based on the intron region of the *Pgk1* gene clock of 0.0051 substitutions per site per MY (million year) [29]. Calibration points were performed using a relaxed uncorrelated lognormal molecular clock. A Yule speciation tree prior was furthermore specified, which assumes a constant speciation rate among lineages, with a log-normal prior for birth rate. MCMC searches were run for 10,000,000 generations under GTR+I model (with the associated parameters specified by ModelTest as the priors). Tracer 1.4 [47] was used to ensure the convergence of the mixing in terms of the effective sample size (ESS) values and the coefficient rate. Resulting trees were analyzed using TreeAnnotator available in BEAST where the burn-in (2000 trees) was removed and a maximum credibility tree was constructed. Trees were then viewed in FigTree v. 1.3.1 (http://tree.bio.ed.ac.uk/).

## Results

### Sequence analysis

Following the screen of *Pgk1* homoeologues, three distinct types of *Pgk1* sequences (St-, P- and Y-type) were obtained from all 15 *Kengyilia* species. At least 15 positive clones (including 5 St-type, 5 P-type and 5 Y-type clones) were sequenced from each accession. In cases when multiple identical sequences resulted from cloned PCR products of one accession, only one sequence was included in the data set. Consequently, 45 unique sequences were obtained and analyzed together with those from 47 diploid taxa representing 18 basic genomes in Triticeae.

**Figure 1:**
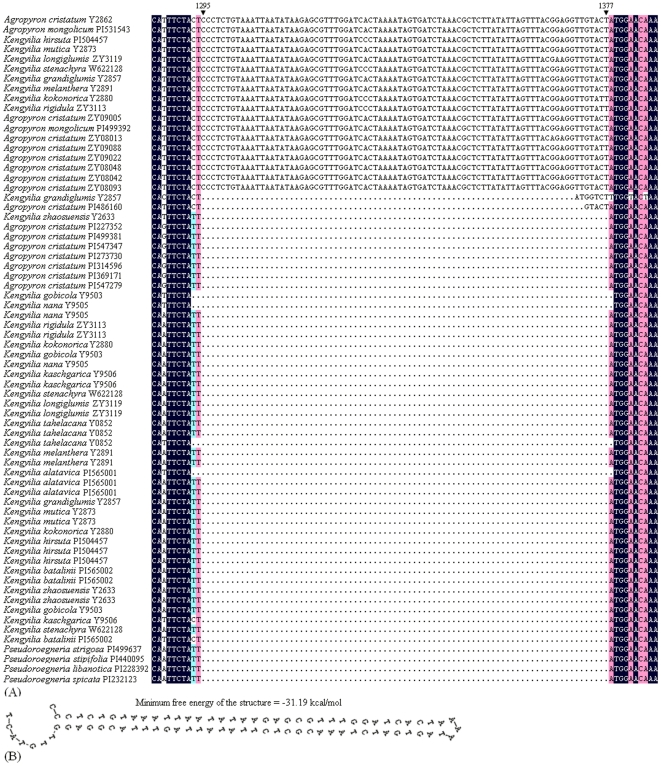
*Pgk1* gene sequence analysis. (**A**) Partial alignment of the amplified sequences of the *Pgk1* gene from *Kengylia* and its putative diploid species. (**B**) Secondary structure of *MITE* elements.

The DNA sequence of the *Pgk1* gene includes 5 exons and 4 introns, which was in agreement with previous studies [29], [30]. The sequence comparison from all the species studied here showed that the length of DNA sequences ranged from 1341 bp to 1484 bp, and the DNA sequences in most accessions were ∼1390 bp. *Pgk1* sequence matrix including both exons and introns contains 1522 characters, of which 20.04% (305/1522) were variable, and 8.74% (133/1522) were parsimony informative. Sequence alignment showed that an 83-bp insertion was detected for the P-type sequences at position 1295–1377 in the intron region from eight *Kengyilia* species (*K. longiglumis*, *K. mutica*, *K. melanthera*, *K. hirsuta*, *K. stenachyra*, *K. rigidula*, *K. kokonorica* and *K. grandiglumis*), two *Agropyron mongolicum* accessions (PI 531543 and PI 499392) and eight *Agropyron cristatum* accessions (Y2862, ZY08013, ZY08042, ZY09022, ZY08048, ZY09088, ZY08093 and ZY09005) (Figure 1, A). Secondary structure analysis indicated an inverted-repeat region in the 83-bp insertion (Figure 1, B). BLAST search against the transposable elements (TEs) stored in the TREP (Triticeae Repeat) showed that the 83-bp insertion belongs to *MITE stowaway* element.

### Phylogenetic analyses

**Figure 2:**
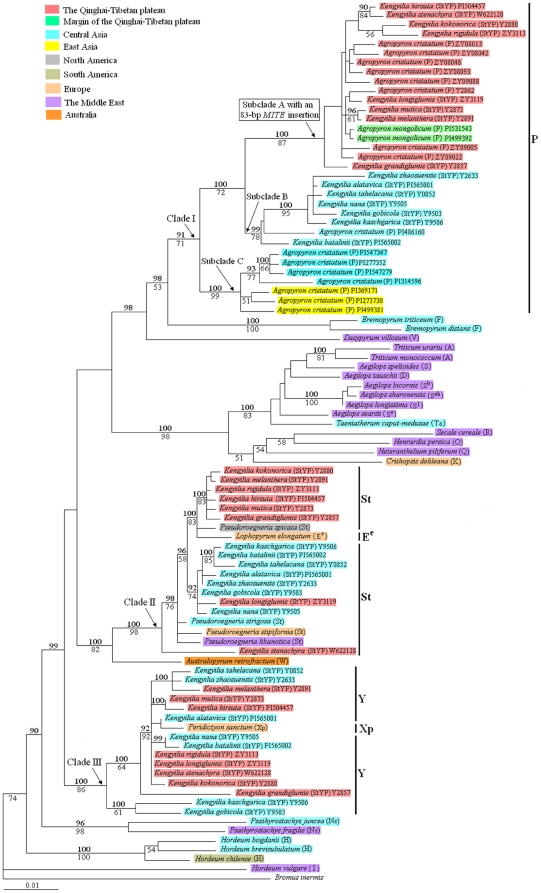
Maximum-likelihood tree inferred form the *Pgk1* sequence of *Kengyila* and its affinitive species within Triticeae. Numbers with bold above nodes are Bayesian posterior probability values ≥90% numbers below nodes are bootstrap values ≥50%. The numbers after species names refer to the distinct homoeologous copy of the *Pgk1* gene. The capital letters in bracket indicate the genome type of the species. Different color labeled the geographic information of *Kengyilia* species and its affinitive donors.

To reveal the putative genome donors of *Kengyilia*, the *Pgk1* sequences of all the polyploid species were included in the phylogenetic analyses (ML and BI), together with 47 diploid taxa representing 18 genomes in Triticeae. ML analysis yielded a single phylogenetic tree (−Lnlikelihood = 9139.1240), with the following estimated ML parameters: the assumed nucleotide frequencies A: 0.2682, C: 0.1918, G: 0.2332, T: 0.3068, the proportion of invariable sites = 0.2519, gamma shape parameter = 0.6693. ML and Bayesian analyses recovered the same topology. The tree illustrated in Figure 2 was the ML tree with posterior probabilities (PP) above and bootstrap support (BS) below branches.

The phylogenetic tree showed that the St-, P- and Y-type sequences from *Kengyilia* species were split into three well supported clades (Figure 2). The Clade I included the P-type sequences of *Kengyilia* and the sequences of *Agropyron* (91% PP and 71% BS). Three subclades (A, B and C subclade) with high statistical support were recognized in this clade. Subclade A included all the *Kengyilia* species and *Agropyron cristatum* accessions from the Qinghai-Tibetan Plateau and *Agropyron mongolicum* from the Alashan (margin of the Qinghai-Tibetan Plateau)(100% PP and 87% BS). It was worth mentioning that the sequence in subclade A had an 83-bp *MITE stowaway* insertion at position 1295–1377. Subclade B contained all the *Kengyilia* species from Central Asia and one *A. cristatum* accession from Central Asia (PI 486160) (99% PP and 78% BS). Subclade C consisted of four *A. cristatum* accessions from Central Asia and three *A. cristatum* accessions from East Asia (100% PP and 99% BS). The Clade II comprised all the St-type sequences of *Kengyilia* and the sequences from *Pseudoroegneria* species and *Lophopyrum elongatum* (100% PP and 98% BS). In this Clade, *Pseudoroegneria spicata* and *Lo. elongatum* were grouped with six *Kengyilia* species from the Qinghai-Tibetan Plateau (100% PP and 83% BS). *Kengyilia longiglumis* from the Qinghai-Tibetan Plateau was clustered with all the sampled *Kengyilia* species from Central Asia (92% PP and 74% BS). Three *Pseudoroegneria* species formed paraphyletic, and *Kengyilia stenachyra* was placed at the base of the Clade II. The Clade III included all the Y-type sequences of *Kengyilia* and the sequence from *Peridictyon sanctum* (100% PP and 86% BS). In Clade III, *Kengyilia kaschgarica* was grouped with *Kengyilia gobicola* with 100% PP and 61% BS, while the remaining *Kengyilia* species formed one subclade (100% PP and 64% BS).

### Nucleotide diversity and strength of selection

Two overall measures of nucleotide diversity, *π* and *θw*, were separately calculated for the St, Y and P genomes of *Kengyilia*, and for *Agropyron* (Table S2). The estimates of nucleotide diversity in the P genome of *Kengyilia* from the Qinghai-Tibetan Plateau were *π* = 0.0105, *θ_w_* = 0.0128, while in the P genome of *Agropyron* from the Qinghai-Tibetan Plateau and its margin region (Alashan), the estimates of nucleotide diversity were *π* = 0.0064, *θ_w_* = 0.0093. The estimates of nucleotide diversity in the P genome of *Kengyilia* from Central Asia were *π* = 0.0088, *θ_w_* = 0.0120, while in the P genome of *Agropyron* from Central Asia, the estimates of nucleotide diversity were *π* = 0.0142, *θ_w_* = 0.0170. *π* was also separately calculated for synonymous and nonsynonymous sites. The overall number of polymorphic sites at homoeologous loci of *Pgk1* sequence from *Kengyilia* St, Y and P genomes was 51, 85, and 82, respectively. The overall number of polymorphic sites in the P genome of *Kengyilia* was lower than that in the P genome of diploid *Agropyron*. The Tajima [34] and Fu and Li's [37] tests were conducted on each of eight data sets (Table S2). Tajima's and Fu and Li's D values of the P genome lineage from the Qinghai-Tibetan Plateau were −0.9473 (P>0.05) and −0.8423 (P>0.05) for *Kengyilia*, and −1.5089 (P<0. 05) and −1.9190 (P<0. 05) for *Agropyron*, respectively. The same parameters in the P genome lineage from Central Asia were −1.5244 (P<0. 05) and −1.5709 (P<0. 05) for *Kengyilia*, and −1.2570 (P<0. 05) and −1.2570 (P<0. 05) for *Agropyron*, respectively.

Speciation genetics suggested that hybridization or differentiation between two species can be inferred through comparisons of shared nucleotide polymorphisms with fixed differences [48]. Closely related taxa are expected to harbor a relative higher level of shared polymorphisms because the divergence event has not lasted long enough to erase all ancestral polymorphisms [36]. The number of shared and fixed differences at *Pgk1* locus between *Kengyilia* and its putative diploid donor were shown in Table 1. Three shared polymorphisms and no fixed difference were observed between the St-type sequence of *Kengyilia* and that of *Pseudoroegneria*. Thirty-four shared polymorphisms and no fixed difference were found between the all the sampled P-type sequence of *Kengyilia* and that of *Agropyron*. For the P genome lineage of sympatric origin, the number of shared polymorphisms was higher than the number of fixed difference, while for the P genome lineage of allopatric origin, the number of shared polymorphisms was lower than the number of fixed difference.

**Table 1:** Estimation of shared polymorphisms and fixed differences between *Kengyilia* and its putative diploid genome donor based on the *Pgk1* sequences.

	S_S_	S_F_
St genome lineage		
*Kengyilia* – *Pseudoroegneria*	3	0
P genome lineage		
*Kengyilia* (QTP^a^) – *Agropyron* (QTP)	6	0
*Kengyilia* (QTP) – *Agropyron* (CA^b^)	5	7
*Kengyilia* (CA) – *Agropyron* (CA)	9	2
*Kengyilia* (CA) – *Agropyron* (QTP)	3	14
*Kengyilia* (Overall) – *Agropyron* (Overall)	34	0

a QTP is the abbreviation of the Qinghai-Tibetan Plateau.

b CA is the abbreviation of Central Asia.

The non-synonymous to synonymous rate ratio d_N_/d_S_ is indicative of the change of selective pressures. The d_N_/d_S_ ratios of >1,  = 1 and <1 indicate positive selection, neutral evolution and purifying selection on the coding portions, respectively. Prior to the estimation of selective constraints on the coding portions of the *Pgk1* gene in *Kengyilia* St, Y and P genome and its putative diploid genome donor, the average non-synonymous (d_N_) and synonymous (d_S_) distances with standard errors were calculated using the modified Nei-Gojobori method (Table S3). Both Z-Test and SLAC statistics showed that almost all the d_N_/d_S_ values were significantly <1, strongly indicating that the *Pgk1* gene in the St, Y and P genomes of *Kengyilia* and its putative diploid genome donor have subjected to purifying selection. Comparison among the *Pgk1* coding portions of *Agropyron* lineages with different geographical region revealed that the d_N_/d_S_ value of the Qinghai-Tibetan Plateau *Agropyron* was not significantly (Z-test with P = 0.1455) below 1 and nearly 3-fold higher than that in the Central Asia *Agropyron*. The McDonald–Kreitman (MK) test of selective pressures was performed to compare the Qinghai-Tibetan Plateau P genome lineage with Central Asia P genome lineage. Total 19 mutations were found in the Qinghai-Tibetan Plateau *Agropyron*, of which 11 were nonsynonymous and eight were synonymous. Among the 19 mutations found in Central Asia *Agropyron*, six were nonsynonymous and 13 were synonymous. Significant departure from neutrality was detected for *Agropyron* (*P* = 0.037) from the Qinghai-Tibetan Plateau.

**Figure 3:**
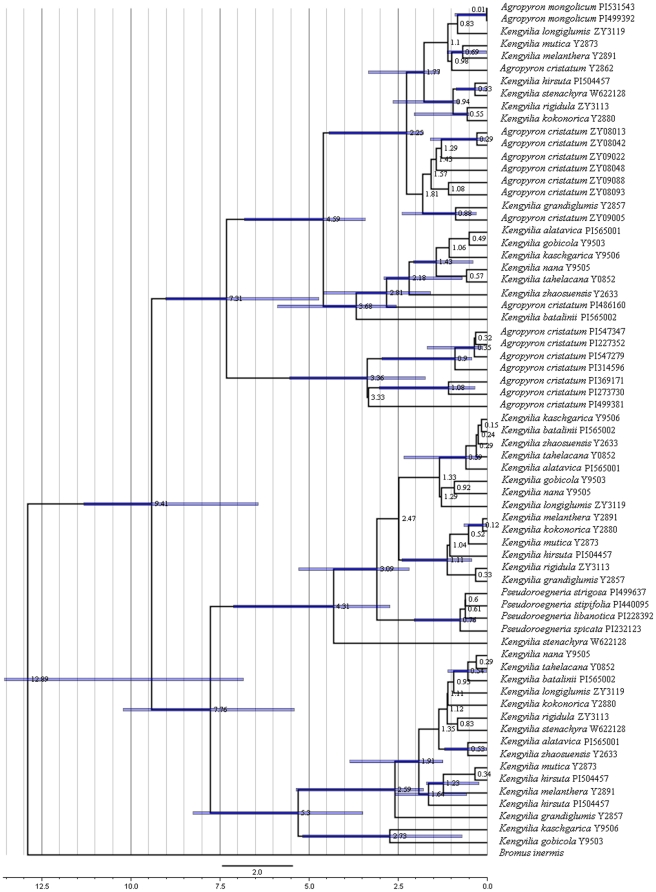
A time-calibrated tree inferred form the intron region of the *Pgk1* sequence of *Kengyila* and its putative donors using a Bayesian relaxed clock method in BEAST.

The BEAST analyses of the intron region of the *Pgk1* sequences from *Kengyilia* and its putative diploid species generated a time-calibrated tree (Figure 3). Under a lognormal relaxed clock, the coefficient of rate variation was estimated to be 0.985 (95% C.I., 0.653–1.352), indicating that relaxed clock was appropriate. The birth rate indicated by the Yule prior is 0.474 (95% C.I., 0.323–0.622). The mean ages with 95% confidence intervals were indicated in the chronogram (Figure 3). Time calibration analysis demonstrated that the divergence time of the St, Y, and P genome lineages was 4.31 MYA (95% C.I., 2.72–7.12), 5.30 MYA (95% C.I., 3.48–8.25), and 7.31 MYA (95% C.I., 4.72–9.01), respectively. The split between the P genome lineages from the Qinghai-Tibetan Plateau and from Central Asia took place about 4.59 MYA (95% C.I., 3.42–6.81).

## Discussion

### *Pgk1* homoeologues and evolutionary history of *Kengyilia*

Cytogenetic analyses have concluded that all the *Kengyilia* species contain the StYP genomic constitutions [15]–[19]. Three homoeologous types of the *Pgk1* gene, St-, Y- and P-type, were obtained from all the polyploid *Kengyilia* species in the present study. Phylogenetic analysis showed that the St-type homoeologous sequences were grouped with the sequences of *Pseudoroegneria* with 100% PP and 98% BS, and the P-type homoeologous sequences were clustered with the sequences of *Agropyron* with 91% PP and 71% BS. Wakeley and Hey [36] pointed out that closely related species are expected to harbor a relative higher level of shared polymorphisms than fixed differences. Our analysis of shared/fixed polymorphisms showed that more shared polymorphisms than fixed differences were observed between the St-type sequences and the sequences of *Pseudoroegneria* and between the P-type sequences and the sequences of *Agropyron*. Phylogenetic and diversity analysis thus indicate that *Kengyilia* is closely related to *Pseudoroegneria* and *Agropyron*. Combined with previous cytogenetic studies [16], [20], it can be concluded that the *Pseudoroegneria* and *Agropyron* species served as the St and P genome donors during the polyploid speciation of the *Kengyilia* species.

The Y genome is represented in all the *Kengyilia* species and many Asiatic tetraploid and some hexaploids in Triticeae [21]. No diploid species containing Y have been identified [49]. Chromosome pairing analysis indicated low affinities between the St and Y genomes [50]. ITS sequence data of Liu et al. [23] showed that the Y genome may be originated from the St genome. *RPB2* [24] and *EF-G* [51] data suggested that the Y genome was sister to the W genome and has a different origin from the St genome. Chromosome pairing suggested that the W genome has very low homoeology with the St and Y genome [52]. Considering the suggestion of Torabinejad and Mueller [52], Sun et al. [51] pointed out that more sequence data are needed to reveal the relationship of the Y genome with other genomes in Triticeae. In this study, phylogenetic analysis indicated that the Y-type *Pgk1* sequences were distinct from the St- and P-type sequences, which provides additional support for the independent origin of the Y genome. In phylogenetic tree (Figure 2), the Y-type sequences were grouped with the sequences from *Peridictyon sanctum* (Xp genome donor). This is in agreement with recent genealogical analysis of single-copy nuclear gene *Acc1* sequence in the species with Y genome in Triticeae (Sha et al., unpublished data), where the Y-type *Acc1* homoeologues were clustered with the sequences from *Dasypyrum* (V genome) species, *Heteranthelium piliferum* (Q genome) and *Peridictyon sanctum* (Xp genome). Therefore, it suggested that the Y genome may be closely related to the Xp genome in *Peridictyon sanctum*.

### Geographical differentiation of P genome

Genus *Agropyron* is the P haplome donor to *Kengyilia* and contains approximately eight diploid (PP) or tetraploid (PPPP) or hexaploid (PPPPPP) species. *Agropyron cristatum* and *A. mongolicum* are the only two diploid species within *Agropyron* [21]. Phytogeographically, *A. cristatum* is widely distributed in Eurasian temperate region, while *A. mongolicum* is restricted in some regions of northern China. The present *Pgk1* gene data showed that the sampled *A. cristatum* from East Asia, the Qinghai-Tibetan plateau, and Central Asia did not form monophyletic group but were scattered into three distinct subclades (subclade A, B, and C). Genetic differentiation among *A. cristatum* population based on pairwise F_ST_ estimates was relatively high, ranging from 14.93% to 65.74% (East Asia – Central Asia: 14.93%; the Qinghai-Tibetan plateau – Central Asia: 51.26%; East Asia – the Qinghai-Tibetan plateau: 65.74%). These results indicated that *A. cristatum* populations from different geographical origins were genetically heterogeneity. High level of genetic differentiation and divergent population structure of *A. cristatum* could be attributed to restricted gene flow caused by geographical isolation.

Data from RAPD [25], RAMP [26], C-banded karyotypes [27], and ITS sequence [28] suggested that *Kengyilia* species were geographical differentiated. Our phylogenetic analysis of *Pgk1* sequences demonstrated that the separation of the P genomes in *Kengyilia* species is in good agreement with their geographical origins – Central Asia and the Qinghai-Tibetan plateau distinction. The accessions of *Kengyilia* species and *A. cristatum* from the Qinghai-Tibetan Plateau, and *A. mongolicum* from Alashan is differentiated from the accessions of *Kengyilia* species and one *A. cristatum* accession from Central Asia. Second, more shared polymorphisms than fixed differences (*Kengyilia* (CA) – *Agropyron* (CA): 9 vs. 2; *Kengyilia* (QTP) – *Agropyron* (QTP): 6 vs. 0) were observed between the sympatric P genome lineage, while less shared polymorphisms than fixed differences (*Kengyilia* (CA) – *Agropyron* (QTP): 3 vs. 14; *Kengyilia* (QTP) – *Agropyron* (CA): 5 vs. 7) were found between the allopatric P genome lineage. Third, an 83 bp *MITE* element insertion in the *Pgk1* gene were found in the P genome of the Qinghai-Tibetan Plateau *Kengyilia* species and their sympatric diploid donors, while this element was absent in the same position of the sequence alignment in the P genome lineages from Central Asia. Finally, time-calibrated phylogeny suggested that speciation event of Central Asia *Kengyilia* species (about 3.68 MYA) may be prior to that of the Qinghai-Tibetan Plateau *Kengyilia* species (about 2.25 MYA). These different lines indicated that the P genome in *Kengyilia* species is highly differentiated according to their geographical origin. The Central Asia and the Qinghai-Tibetan Plateau *Kengyilia* species thus have independent origins.

Recent studies using genetic markers in many genera suggested that multiple origins (including independent origin) of polyploid species are the rule rather than the exception [2], [53]. A better understanding of the potential evolutionary outcomes for polyploid populations of independent origin is of particular evolutionary interest. Symonds et al. [53] emphasized that the fates of polyploid populations of independent origins varied depending on the amount of genetic variation initially contributed by the diploid progenitors. The present *Pgk1* gene genealogical structure and patterns of shared/fixed polymorphisms indicated the occurrence of independent origins of *Kengyilia* species. This offers an opportunity to address the potential evolutionary outcomes of independent origins within *Kengyilia*. On the basis of *Pgk1* sequences of the P genome lineage from the Qinghai-Tibetan Plateau, the level of nucleotide diversity in *Kengyilia* (*π* = 0.0105; *θ*_w_ = 0.0128) was higher than that in diploid *Agropyron* (*π* = 0.0064; *θ*_w_ = 0.0093), and Tajima's and Fu and Li's D statistic suggests a departure from the equilibrium neutral model at this locus, with an excess of rare sequence variants in *Kengyilia* species. Greater diversity could reflect gene flow from diploid *Agropyron* population. For the P genome lineage from Central Asia, the level of nucleotide diversity in *Kengyilia* (*π* = 0.0088; *θ*_w_ = 0.0120) was lower than that in diploid *Agropyron* (*π* = 0.0142; *θ*_w_ = 0.0170), and the values of Tajima's and Fu and Li's D statistic in *Kengyilia* were significantly negative and lower than the values of same parameter calculated from *Agropyron*. This is compatible with a genetic bottleneck created by recent polyploidization. Diverged levels of nucleotide suggested that the P genome lineages of *Kengyilia* with independent origin have distinct evolutionary potentials.

### Evolution of Pgk1 sequences in *Kengyilia*

Since gene duplication results in functional redundancy, divergent selective pressure may act on the duplicated copies that are critical for the subsequent variation, retention or loss of the duplicated genes [54]. Isolation and characterization of three divergent *Pgk1* homoeologues from all the hexaploid *Kengyilia* species studied here suggested the retention of triplicated *Pgk1* homoeologues in *Kengyilia* species. The d_N_/d_S_ ratio of three divergent *Pgk1* homoeologues was significantly below 1 (Z-test with P<0.05; SLAC with 95% C.I.: 0–1), suggesting that the *Pgk1* sequences are selectively constrained as most mutations in functional genes are expected to be disadvantageous.

The d_N_/d_S_ value of the Qinghai-Tibetan Plateau *Agropyron* was nearly 3-fold higher than that in Central Asia *Agropyron*. Significant MK test (*P* = 0.037) between them suggested an excess of nonsynonymous substitutions, which is traditional viewed as an outcome of positive selection. This appears paradoxical in light of the strong signature of purifying selection in *Pgk1* sequences. Relaxed purifying selection, selective sweep, and population expansion may explain this paradox. The relaxed purifying selection hypothesis is very unlikely because it did not explain our result of less significant Tajima's D values in the high d_N_/d_S_ group (P = 0.044) than in the low d_N_/d_S_ group (P = 0.017). The deficiency of synonymous polymorphisms in the high d_N_/d_S_ group in comparison with the low d_N_/d_S_ group was also not the result of relaxed purifying selection. The following evidences support the hypothesis of selective sweep. First, the d_N_/d_S_ value of the Qinghai-Tibetan Plateau *Agropyron* was significantly higher than that in Central Asia *Agropyron*. Significant difference in d_N_/d_S_ ratio was considered to be a result of selective sweep [55]. Second, our result showed less levels of nucleotide diversity in the high d_N_/d_S_ group than in the low d_N_/d_S_ group, which is consistent with the expectation that sweep results in reduced polymorphism in the high d_N_/d_S_ group [55]. Third, Palmé et al. [56] emphasized that selective sweep would cause more negative Tajima's D and lower silent diversity in the high d_N_/d_S_ group than in the low d_N_/d_S_ group. Corresponding to this suggestion, the present result showed that the Tajima's D value in the high d_N_/d_S_ group (−1.5089) is more negative than that in the low d_N_/d_S_ group (−1.2570), and the levels of silent diversity in the high d_N_/d_S_ group (*π* = 0.0089) is less than that in the low d_N_/d_S_ group (*π* = 0.0236). Finally, given that the *Pgk1* gene in the Qinghai-Tibetan Plateau *Agropyron* population has undergone selective sweep, it is suggested that the sweep event for the *Pgk1* gene in Plateau may be associated with an evolutionary adaptation to local cold climate conditions. Recent study focusing on the responses of plant to cold stress has revealed that Phosphoglycerate kinase (Pgk1) is an up-regulated response protein to cold stress, a feature of adaptive evolution [57]. Palaeoclimatic evidence indicated that the cold climate effects of the Qinghai-Tibetan Plateau resulted from its large-scale uplifting and consequent glacial cycles during the Quaternary (2.4 MYA to the present) [58], [59]. The present molecular dating suggested that the divergence time of the P genome lineage including the high d_N_/d_S_ *Agropyron* population from the Qinghai-Tibetan Plateau was dated to 2.25 MYA, and the divergence time of the P genome lineage including the low d_N_/d_S_ *Agropyron* population from Central Asia was dated to 3.38 MYA. Considering the conversion of low to high d_N_/d_S_ value resulting from selective sweep, the age of selective sweep might have happened ≈2.3–3.4 MYA. Therefore, it is possible that a relatively long-time sweep event allow the *Pgk1* gene within *Agropyron* to adapt to cold climate triggered by the recent uplifts of the Qinghai-Tibetan Plateau. Because demographic processes such as range expansion can have a similar impact on DNA variation to that caused by a selective sweep in a population [60]. It can also not rule out the possibility that population expansion might have contributed to the present significant MK tests and difference in the d_N_/d_S_ ratio. Analysis of diversity showed that the levels of diversity in the Qinghai-Tibetan Plateau *Agropyron* population (*π* = 0.0089; *θw* = 0.0093) is significant lower than that in Central Asia population (*π* = 0.0236; *θw* = 0.0170), indicating a recent expansion in the Qinghai-Tibetan Plateau *Agropyron* population. This suggestion is further determinative of the more significant negative Tajima's and Fu and Li's D values. Population expansion in associated with Pleistocene glacial cycles might have accelerated the fixation of mildly deleterious replacement mutations that became effectively neutral in the Qinghai-Tibetan Plateau *Agropyron* populations.

Duplicate genes in polyploid lineages are often preserved in function by more strongly purifying selection [61]. Comparative analyses showed that the d_N_/d_S_ value in the P genome of the Qinghai-Tibetan Plateau *Kengyilia* species was lower than that in their sympatric diploid relatives, indicating that more strongly purifying selection acts to conserve the function of the *Pgk1* gene in *Kengyilia*. However, a slightly elevated d_N_/d_S_ value in the P genome of the Central Asia *Kengyilia* species compared to their sympatric diploid relatives might suffer from polyploidization bottleneck as suggested by the present estimate of diversity, because population bottleneck may result in reduced selection [62].

It is worth mentioning that the d_N_/d_S_ value of the P genome of *Kengyilia* lineage from the Qinghai-Tibetan Plateau (d_N_/d_S_ = 0.2645) was higher than that from Central Asia (d_N_/d_S_ = 0.2018). Non-significantly negative Tajima's D value coupled with greater diversity in the high d_N_/d_S_ group excluded the possibility that selective sweep could resulted in the difference in the d_N_/d_S_ value of the P genome of *Kengyilia*. A highly non-significant difference between this two d_N_/d_S_ ratios (*P* = 0.499 by Fisher's exact test) based on the McDonald-Kreitman test was found, rejecting the hypothesis of relaxed purifying selection. Considering the difference in the d_N_/d_S_ value of two allopatric *Agropyron* lineages, it is possible that the difference in the d_N_/d_S_ value of the P genome of two allopatric *Kengyilia* lineages may be genetically from geographically differentiated P genome donors via independent origins. This difference is not completely erased, although polyploidization bottleneck might occur in Central Asia *Kengyilia* lineages and strong purifying selection might act on the *Pgk1* gene in the Qinghai-Tibetan Plateau *Kengyilia* lineages.

## Supporting Information

Table S1 ***Kengyilia*** **species and other related genera in Triticeae used in this study.**
(DOC)

Table S2 **Estimates of nucleotide diversity and test statistics at** ***Pgk1*** **locus in** ***Kengyilia*** **St, Y and P genome and its putative diploid genome donor.**
(DOC)

Table S3 **Detection of selection pressure on the coding portions of the** ***Pgk1*** **gene in** ***Kengyilia*** **St, Y and P genome and its putative diploid genome donor.**
(DOC)
