# Supplementary material for: Evolutionary Dynamics of the Pgk1 Gene in the Polyploid Genus Kengyilia (Triticeae: Poaceae) and Its Diploid Relatives
Source: PLoS One. 2012 Feb 20;7(2):e31122. doi: 10.1371/journal.pone.0031122 (PMC3282717; doi:10.1371/journal.pone.0031122)
Supplement: Table S1 — Kengyilia species and other related genera in Triticeae used in this study. (DOC) [file pone.0031122.s001.doc]

Table S1 *Kengyilia* species and other related genera in Triticeae used in this study

| Taxa | Accession No. | Genome (ploidy) | Origin | GenBank Accession No. |
| --- | --- | --- | --- | --- |
| *Aegilops* L. |  |  |  |  |
| *Aegilops bicornis* | ND | **S^b^** (2×) | The Middle East | **AF343485** |
| *Aegilops longissima* | ND | **S^l^** (2×) | The Middle East | **AF343487** |
| *Aegilops searsii* | ND | **S^s^** (2×) | The Middle East | **AF343489** |
| *Aegilops sharonensis* | ND | **S^sh^** (2×) | The Middle East | **AF343486** |
| *Aegilops speltoides* | ND | **S** (2×) | The Middle East | **AF343491** |
| *Aegilops tauschii* | ND | **D** (2×) | The Middle East | **AF343479** |
| *Agropyron* Gaertn. |  |  |  |  |
| *Agropyron cristatum* | PI 277352 | **P** (2×) | Russian Federation | FJ711023 |
| *Agropyron cristatum* | PI 486160 | **P** (2×) | Kazakstan | JF965622 |
| *Agropyron cristatum* | PI 547347 | **P** (2×) | Urumqi, Xinjiang, China | JF965620 |
| *Agropyron cristatum* | PI 314596 | **P** (2×) | Alma Ata, Kazakastan | JF965621 |
| *Agropyron cristatum* | PI 273730 | **P** (2×) | Buryatia, Russian Federation | JF965625 |
| *Agropyron cristatum* | PI 369171 | **P** (2×) | Yakutsk, Russian Federation | JF965623 |
| *Agropyron cristatum* | PI 499381 | **P** (2×) | Xilinhot, Inner Mongolia, China | JF965619 |
| *Agropyron cristatum* | PI 547279 | **P** (2×) | Orenburg, Russian Federation | JF965624 |
| *Agropyron cristatum* | ZY 09088 | **P** (2×) | Dulan, Qinghai, China | JF965630 |
| *Agropyron cristatum* | ZY 08013 | **P** (2×) | Xining, Qinghai, China | JF965629 |
| *Agropyron cristatum* | ZY 09022 | **P** (2×) | Gansu, China | JF965631 |
| *Agropyron cristatum* | ZY 09005 | **P** (2×) | Sunan, Gansu, China | JF965628 |
| *Agropyron cristatum* | ZY 08093 | **P** (2×) | Xinhai, Qinghai, China | JF965634 |
| *Agropyron cristatum* | ZY 08042 | **P** (2×) | Qinghaihu, Qinghai, China | JF965633 |
| *Agropyron cristatum* | Y 2862 | **P** (2×) | Qilian, Qinghai, China | JF965626 |
| *Agropyron cristatum* | ZY 08048 | **P** (2×) | Guomaying, Qinghai, China | JF965632 |
| Taxa | Accession No. | Genome (ploidy) | Origin | GenBank Accession No. |
| *Agropyron monglicum* | PI 531543 | **P** (2×) | Inner Mongolia, China | JF965627 |
| *Agropyron monglicum* | PI 499392 | **P** (2×) | Inner Monggol, China | FJ711024 |
| *Australopyrum* (Tsvelev.) Á. Löve |  |  |  |  |
| *Australopyrum retrofractum* | PI 533013 | **W** (2×) | New South Wales, Australia | FJ711025 |
| *Crithopsis* (Schult.) Roshev. |  |  |  |  |
| *Crithopsis delileana* | ND | **K** (2×) | Greece | FJ711026 |
| *Dasypyrum* (L.) Candargy |  |  |  |  |
| *Dasypyrum villosum* | PI 251478 | **V** (2×) | Turkey | FJ711027 |
| *Eremopyrum* Jaub. et Spach. |  |  |  |  |
| *Eremopyrum distans* | TA 2229 | **F** (2×) | Afghanistun | FJ711018 |
| *Eremopyrum triticeum* | Y 206 | **F** (2×) | Xinjiang, China | FJ711028 |
| *Henrardia* C. E. Hubb. |  |  |  |  |
| *Henrardia persica* | PI 401349 | **O** (2×) | Turkey | FJ711029 |
| *Heteranthelium* Hochst. |  |  |  |  |
| *Heteranthelium piliferum* | PI 401351 | **Q** (2×) | Iran | FJ711030 |
| *Hordeum* Linn. |  |  |  |  |
| *Hordeum bogdanii* | PI 531761 | **H** (2×) | Xinjiang, China | FJ711020 |
| *Hordeum chilense* | PI 531781 | **H** (2×) | Chile | FJ711017 |
| *Hordeum brevisubulatum* |  |  |  | FJ711019 |
| *Hordeum vulgare* | Betzes | **I** (2×) | The Middle East | **AF343494** |
| *Lophopyrum* (Host) Á. Löve |  |  |  |  |
| *Lophopyrum elongatum* | PI 531719 | **E^e^** (2×) | St. Angulf, France | FJ711035 |
| *Peridictyon* O. Seber, S. Frederiksen & C. Baden |  |  |  |  |
| *Peridictyon sanctum* | H 3841 | **Xp** (2×) | Greece | FJ711037 |
| *Psathyrostachys* Nevski |  |  |  |  |
| Taxa | Accession No. | Genome (ploidy) | Origin | GenBank Accession No. |
| *Psathyrostachys fragilis* | Y 882 | **Ns** (2×) | Iran | FJ711016 |
| *Psathyrostachys juncea* | PI 222050 | **Ns** (2×) | Afghanistun | FJ711031 |
| *Pseudoroegneria* (Nevski) Á. Löve |  |  |  |  |
| *Pseudoroegneria libanotica* | PI 228392 | **St** (2×) | Iran | FJ711032 |
| *Pseudoroegneria spicata* | PI 232123 | **St** (2×) | Washington, USA | FJ711015 |
| *Pseudoroegneria stipifornia* | PI 440095 | **St** (2×) | Yankulskaya, Russian | FJ711033 |
| *Pseudoroegneria strigosa* | PI 499637 | **St** (2×) | Xinjiang, China | FJ711034 |
| *Secale* L. |  |  |  |  |
| *Secale cereale* | Imperial | **R** (2×) | The Middle East | **AF343493** |
| *Taeniatherum* (L.) Nevski |  |  |  |  |
| *Taeniatherum caput-medusae* | PI 220591 | **Ta** (2×) | Afghanistan | FJ711021 |
| *Triticum* L. |  |  |  |  |
| *Triticum monococcum* | TA 2025 | **A^m^** (2×) | The Middle East | FJ711022 |
| *Triticum urartu* | TA 763 | **A** (2×) | Lebanon | **AF343474** |
| *Kengyilia* Yen et J. L. Yang |  |  |  |  |
| *Kengyilia alatavica* | PI 565001 | **StYP (**6×**)** | Kazakhstan | JF965588, JF965589, JF965590 |
| *Kengyiia batalinii* | PI 565002 | **StYP (**6×**)** | Kazakhstan | JF965582, JF965583, JF965584 |
| *Kengyilia gobicola* | Y 9503 | **StYP (**6×**)** | Taxkorgan, Xinjiang, China | JF965573, JF965574, JF965575 |
| *Kengyilia grandiglumis* | Y 2857 | **StYP (**6×**)** | Haiyan, Qinghai, China | JF965603, JF965604, JF965605 |
| *Kengyilia hirsuta* | PI 504457 | **StYP (**6×**)** | Qinhai Lake, Qinghai, China | JF965609, JF965610, JF965611 |
| *Kengyilia kaschgarica* | Y 9506 | **StYP (**6×**)** | Xinjiang, China | JF965579, JF965580, JF965581 |
| *Kengyilia kokonorica* | Y 2880 | **StYP (**6×**)** | Gonghe, Qinghai, China | JF965613, JF965614, JF965615 |
| *Kengyilia longiglumis* | ZY 3119 | **StYP (**6×**)** | Xiahe, Gansu, China | JF965594, JF965595, JF965596 |
| *Kengyilia melanthera* | Y 2891 | **StYP (**6×**)** | Maduo, Qinghai, China | JF965600, JF965601, JF965602 |
| *Kengyilia mutica* | Y 2873 | **StYP (**6×**)** | Geermu, Qinghai, China | JF965576, JF965577, JF965578 |
| Taxa | Accession No. | Genome (ploidy) | Origin | GenBank Accession No. |
| *Kengyilia nana* | Y 9505 | **StYP (**6×**)** | Taxkorgan, Xinjiang, China | JF965597, JF965598, JF965599 |
| *Kengyilia rigidula* | ZY 3113 | **StYP (**6×**)** | Xiahe, Gansu, China | JF965606, JF965607, JF965608 |
| *Kengyilia stenachyra* | W6 22128 | **StYP (**6×**)** | Xiahe, Gansu, China | JF965616, JF965617, JF965618 |
| *Kengyilia tahelacana* | Y 0852 | **StYP (**6×**)** | Wensu, Xinjiang, China | JF965585, JF965586, JF965587 |
| *Kengyilia zhaosuensis* | Y 2633 | **StYP (**6×**)** | Zhaosu, Xinjiang, China | JF965591, JF965592, JF965593 |
| *Bromus* L. |  |  |  |  |
| *Bromus inermis* | PI618974 | ND | Xinjiang, China | FJ711014 |

The underlined GenBank accession numbers with bold represent previously published sequences from the GenBank ([http://www.ncbi.nlm.nih.gov](http://www.ncbi.nlm.nih.gov/)). ND: not determined.
