# Supplementary material for: Evolutionary Dynamics of the Pgk1 Gene in the Polyploid Genus Kengyilia (Triticeae: Poaceae) and Its Diploid Relatives
Source: PLoS One. 2012 Feb 20;7(2):e31122. doi: 10.1371/journal.pone.0031122 (PMC3282717; doi:10.1371/journal.pone.0031122)
Supplement: Table S2 — Estimates of nucleotide diversity and test statistics at Pgk1 locus in Kengyilia St, Y and P genome and its putative diploid genome donor. (DOC) [file pone.0031122.s002.doc]

Table S2 Estimates of nucleotide diversity and test statistics at *Pgk1* locus in *Kengyilia* St, Y and P genome and its putative diploid genome donor

|  | *n* | *s* | *π*_overall_ | *π_synonymous_* | *π*_non-_*_synonymous_* | *θ_w_* | Fu & Li’s D | Tajima’s D |
| --- | --- | --- | --- | --- | --- | --- | --- | --- |
| *Kengyilia* |  |  |  |  |  |  |  |  |
| St | 1359 | 51 | 0.0076 | 0.0167 | 0.0030 | 0.0115 | -2.2019 (P<0.05) | -1.4726 (P<0.05) |
| Y | 1362 | 85 | 0.0106 | 0.0107 | 0.0027 | 0.0188 | -2.2987 (P<0.05) | -1.8786 (P<0.05) |
| P^a^ (QTP) | 1451 | 48 | 0.0105 | 0.0146 | 0.0030 | 0.0128 | -0.8423 (P>0.05) | -0.9473 (P>0.05) |
| P^b^ (CA) | 1358 | 40 | 0.0088 | 0.0139 | 0.0021 | 0.0120 | -1.5709 (P<0.05) | -1.5244 (P<0.05) |
| P^c^ (Overall) | 1356 | 82 | 0.0186 | 0.0348 | 0.0028 | 0.0186 | -0.5641 (P>0.05) | -0.0032 (P>0.05) |
| *Agropyron* |  |  |  |  |  |  |  |  |
| P^a^ (QTP) | 1366 | 36 | 0.0064 | 0.0089 | 0.0038 | 0.0093 | -1.9190 (P<0.05) | -1.5089 (P<0.05) |
| P^b^ (CA) | 1355 | 48 | 0.0142 | 0.0236 | 0.0036 | 0.0170 | -1.2570 (P<0.05) | -1.2570 (P<0.05) |
| P^d^ (Overall) | 1346 | 108 | 0.0209 | 0.0437 | 0.0039 | 0.0233 | -1.6744 (P>0.05) | -0.4432 (P>0.05) |

The n is the number of the sites (excluding sites with gaps/missing data), *s* is the number of segregating sites, *π* is the average pairwise diversity, and *θw* is the diversity based on the number of segregating sites.

a Nucleotide diversity and test statistics of *Pgk1* sequences were based on the P genome lineage from the Qinghai-Tibetan Plateau (QTP).

b Nucleotide diversity and test statistics of *Pgk1* sequences were based on the P genome lineage from Central Asia (CA).

c Nucleotide diversity and test statistics of *Pgk1* sequences were based on all sampled P genome lineage in *Kengyilia*.

d Nucleotide diversity and test statistics of *Pgk1* sequences were based on all sampled P genome lineage in *Agropyron*.
