# Supplementary material for: Evolutionary Dynamics of the Pgk1 Gene in the Polyploid Genus Kengyilia (Triticeae: Poaceae) and Its Diploid Relatives
Source: PLoS One. 2012 Feb 20;7(2):e31122. doi: 10.1371/journal.pone.0031122 (PMC3282717; doi:10.1371/journal.pone.0031122)
Supplement: Table S3 — Detection of selection pressure on the coding portions of the Pgk1 gene in Kengyilia St, Y and P genome and its putative diploid genome donor. (DOC) [file pone.0031122.s003.doc]

Table S3 Detection of selection pressure on the coding portions of the *Pgk1* gene in *Kengyilia* St, Y and P genome and its putative diploid genome donor

|  | d_S_ | d_N_ | d_N_/d_S_ | Z-Test of purifying selection | SLAC (95% C.I.) |
| --- | --- | --- | --- | --- | --- |
| *Kengyilia* |  |  |  |  |  |
| St | 0.0137 ± 0.0035 | 0.0033 ± 0.0011 | 0.2408 | P = 0.0031 (d_S_-d_N_ = 2.7892) | d_N_/d_S_ = 0.2238 (0.1162, 0.3835) |
| Y | 0.0088 ± 0.0020 | 0.0028 ± 0.0009 | 0.3182 | P = 0.0063 (d_S_-d_N_ = 2.5304) | d_N_/d_S_ = 0.2484 (0.1330, 0.4168) |
| P^a^ (QTP) | 0.0121 ± 0.0039 | 0.0032 ± 0.0013 | 0.2645 | P = 0.0168 (d_S_-d_N_ = 2.1494) | d_N_/d_S_ = 0.2626 (0.1128, 0.5082) |
| P^b^ (CA) | 0.0114 ± 0.0035 | 0.0023 ± 0.0010 | 0.2018 | P = 0.0069 (d_S_-d_N_ = 2.4998) | d_N_/d_S_ = 0.2130 (0.0763, 0.4580) |
| *Agropyron* |  |  |  |  |  |
| P^a^ (QTP) | 0.0073 ± 0.0027 | 0.0041± 0.0012 | 0.5616 | P = 0.1455 (d_S_-d_N_ = 1.0600) | d_N_/d_S_ = 0.6478 (0.3362, 1.1103) |
| P^b^ (CA) | 0.0195 ± 0.0051 | 0.0038 ± 0.0015 | 0.1950 | P = 0.0022 (d_S_-d_N_ = 2.9080) | d_N_/d_S_ = 0.1999 (0.0794, 0.4056) |

a Detection of selection pressure on the coding portions of the *Pgk1* gene were based on the P genome lineage from the Qinghai-Tibetan Plateau (QTP).

b Detection of selection pressure on the coding portions of the *Pgk1* gene were based on the P genome lineage from central Asia (CA).
